# Improved Tumor Control Following Radiosensitization with Ultrasound-Sensitive Oxygen Microbubbles and Tumor Mitochondrial Respiration Inhibitors in a Preclinical Model of Head and Neck Cancer

**DOI:** 10.3390/pharmaceutics15041302

**Published:** 2023-04-21

**Authors:** Quezia Lacerda, Hebah Falatah, Ji-Bin Liu, Corinne E. Wessner, Brian Oeffinger, Ankit Rochani, Dennis B. Leeper, Flemming Forsberg, Joseph M. Curry, Gagan Kaushal, Scott W. Keith, Patrick O’Kane, Margaret A. Wheatley, John R. Eisenbrey

**Affiliations:** 1 Department of Radiology, Thomas Jefferson University, Philadelphia, PA 19107, USA; 2 School of Biomedical Engineering, Science and Health Systems Drexel University, Philadelphia, PA 19104, USA; 3 College of Applied Medical Sciences, King Saud Bin Abdulaziz University for Health Sciences, Jeddah 22384, Saudi Arabia; 4 King Abdullah International Medical Research Center, Jeddah 22384, Saudi Arabia; 5 Department of Pharmaceutical Sciences, Thomas Jefferson University, Philadelphia, PA 19107, USA; 6 Department of Pharmaceutical Sciences, Wegmans School of Pharmacy, St. John Fisher University, Rochester, NY 14618, USA; 7 Department of Radiation Oncology, Thomas Jefferson University, Philadelphia, PA 19107, USA; 8 Department of Otolaryngology, Thomas Jefferson University, Philadelphia, PA 19107, USA; 9 Division of Biostatistics, Department of Pharmacology, Physiology, and Cancer Biology, Thomas Jefferson University, Philadelphia, PA 19107, USA

**Keywords:** radiotherapy, tumor hypoxia, contrast-enhanced ultrasound, oxygen-loaded microbubbles, drug delivery, head and neck cancer, lonidamine, metformin

## Abstract

Tumor hypoxia (oxygen deficiency) is a major contributor to radiotherapy resistance. Ultrasound-sensitive microbubbles containing oxygen have been explored as a mechanism for overcoming tumor hypoxia locally prior to radiotherapy. Previously, our group demonstrated the ability to encapsulate and deliver a pharmacological inhibitor of tumor mitochondrial respiration (lonidamine (LND)), which resulted in ultrasound-sensitive microbubbles loaded with O_2_ and LND providing prolonged oxygenation relative to oxygenated microbubbles alone. This follow-up study aimed to evaluate the therapeutic response to radiation following the administration of oxygen microbubbles combined with tumor mitochondrial respiration inhibitors in a head and neck squamous cell carcinoma (HNSCC) tumor model. The influences of different radiation dose rates and treatment combinations were also explored. The results demonstrated that the co-delivery of O_2_ and LND successfully sensitized HNSCC tumors to radiation, and this was also enhanced with oral metformin, significantly slowing tumor growth relative to unsensitized controls (*p* < 0.01). Microbubble sensitization was also shown to improve overall animal survival. Importantly, effects were found to be radiation dose-rate-dependent, reflecting the transient nature of tumor oxygenation.

## 1. Introduction

Radiation therapy remains a preferred treatment approach in patients with head and neck squamous cell carcinoma (HNSCC), with nearly 75% receiving radiation therapy [1]. HNSCC development has historically been caused by tobacco use and excessive alcohol consumption. However, in recent decades, human papillomavirus (HPV) infection has become an increasingly recognized causative factor for oropharyngeal tumors (80%), and HNSCC has become the most common HPV-related malignancy [2,3,4]. The radiation dose is often limited by the tolerance of the associated normal tissue located in the radiation field. The conventional clinical radiation dose for HNSCC consists of fractionated doses of 1.5–2 Gy of external-beam radiation five times a week for up to seven weeks [5,6,7]. Roughly two-thirds of the biological damage produced by X-rays is caused by free radicals from the reactive oxygen species (ROS) induced by ionizing radiation. Furthermore, the damage created by free radicals in DNA can be more readily repaired in a hypoxic environment [8,9].

Tumor hypoxia has been identified as the key mechanism of radiation therapy resistance in tumors due to the limited production of ROS. The irregular, angiogenic vasculature that forms in tumors cannot supply sufficient oxygen to the rapidly dividing cells, resulting in an oxygen consumption rate in tumor cells that is greater than the amount of oxygen supplied by the blood flow in these tumors [8,10,11]. Healthy tissue generally exhibits oxygen partial pressures (pO_2_) ranging from 40 to 60 mmHg, while many tumors exhibit partial pressures between 2 and 18 mmHg [12]. Several studies have shown that tumor hypoxia is directly associated with a poor prognosis in patients with advanced HNSCC [13,14,15]. Molecular oxygen is a well-known radiosensitizer, and studies have shown that relatively low levels (<20 mmHg) of oxygen are required for radiosensitization [10,16].

To overcome hypoxia-associated radiotherapy resistance, systemic approaches have been investigated to improve tumor energetics and oxygenation prior to radiotherapy. Recent studies explored the ability to overcome hypoxia in solid tumors prior to photodynamic therapy using nanoparticles delivered along with mitochondria-associated oxidative phosphorylation disrupting drugs [17,18,19]. One such drug, metformin, is a biguanide that is generally used for the treatment of type 2 diabetes and has been shown to reduce cancer-related morbidity and mortality. Metformin is an attractive adjunct given its 50–60% absolute oral bioavailability while also inhibiting mitochondrial respiration [20,21]. However, this and other systemic approaches lack tumor specificity. Consequently, more recent work has focused on localized approaches for overcoming tumor hypoxia, including ultrasound-triggered delivery of bioactive drugs and gases prior to radiotherapy [22].

Our group has demonstrated the effectiveness of using surfactant-shelled microbubbles with oxygen cores (SE61O_2_) to deliver oxygen to hypoxic tumors prior to radiation therapy [6,23,24,25,26]. The feasibility of this approach was first demonstrated in breast tumor xenografts, where oxygenation significantly improved both tumor control and animal survival (*p* < 0.03) [24]. However, that study’s limitation was that the duration of oxygenation (less than 3 min) hindered clinical adoption. In an effort to prolong oxygenation, we recently showed the ability to deliver both O_2_ and lonidamine (LND), a pharmacological mitochondrial respiration inhibitor, in an HNSCC tumor model, which prolonged the duration of tumor oxygenation for up to 5 min [6]. Furthermore, when investigating LND bioavailability, SE61 proved to be an optimal targeted delivery vehicle, improving LND biodistribution in tissue and plasma.

Building on this prior work, the study presented here aimed to determine whether the combination of O_2_ microbubbles with mitochondrial respiration inhibitors (both co-encapsulated LND and oral metformin) would improve tumor treatment response and survival by improving radiosensitization. LND has been shown to not only inhibit aerobic glycolysis in tumor cells but also increase aerobic glycolysis in normal cells and display a selective effect on tumors by producing intracellular acidosis [27,28]. Additionally, given the limited duration of oxygenation with this platform, the influence of radiation dose rate was also explored in this model. These are essential steps in understanding the mechanisms of radiotherapy sensitization and demonstrating suitability for clinical translation.

## 2. Materials and Methods

### 2.1. Microbubble Fabrication

Surfactant-shelled oxygen microbubbles (SE61O_2_) were fabricated in accordance with our previously reported methods [6]. Briefly, LND (Sigma-Aldrich; St. Louis, MO, USA) was incubated with water-soluble vitamin E (Tocopheryl polyethylene glycol succinate; TPGS) (Eastman Chemical Company; Kingsport, TN, USA) micelles while continuously stirring for 48 h at 37 °C. The micelle solution (with or without LND) was mixed with sorbitan monostearate (Montane 60 PHA Premium), a gift from Seppic (Paris, France), after being autoclaved for 35 min. The mixture of the two surfactants was then purged with perfluorocarbon (PFC) gas for one minute (octafluoropropane, Advanced Specialty Gasses; Reno, NV, USA), followed by sonication while continuously purging with PFC gas. The resultant microbubbles were separated using gravity. After washing (to separate the unincorporated surfactant), the microbubbles were collected and diluted 1:1 (*v/v*) with 10% (*w/v*) glucose to provide lyoprotection. The microbubbles were then transferred in 2 mL aliquots into 10 mL lyophilization vials. Samples were flash frozen in a −20 °C bath, lyophilized using a freeze-dryer under vacuum for at least 20 h, and capped under vacuum. Prior to use, microbubbles were charged with a sterile-filtered (0.2 μm filter) gas of choice, PFC, oxygen, or nitrogen (Airgas, Radnor, PA, USA) through the vial stoppers using a syringe needle and reconstituted immediately by injecting 2 mL of 0.5 X phosphate-buffered saline (PBS).

### 2.2. In Vitro Acoustic Characterization

Acoustic enhancement and stability were quantified in vitro in a closed-loop flow phantom setup (ATS Laboratories, CIRS, Norfolk, VA, USA). Microbubbles were injected and insonated using a 10L4 transducer and an Acuson Sequoia ultrasound scanner (Siemens Healthineers, Issaquah, WA, USA) at room temperature. Imaging of the microbubbles flowing through the embedded vessel was performed in cadence pulse sequencing mode every 30 s for 10 min (n = 3, for microbubbles charged with O_2_ and N_2_ with and without LND) at low mechanical index (MI) imaging to visualize enhancement (MI = 0.12). The mean enhancement returned to the transducer was determined by drawing regions of interest on the contrast imaging plane; all analyses were performed in ImageJ (NIH, Bethesda, MD, USA). Enhancement (in dB) was determined every 30 s and plotted against time.

### 2.3. Light Microscopy

To access visual changes to microbubble physical characteristics, an Olympus 1X71 microscope (Olympus Corporation, Tokyo, Japan) was used to image drug-loaded and -unloaded SE61. Images were processed using the Olympus cellSens Standard software (Olympus Corporation, Tokyo, Japan). Images were obtained using a magnification factor of 64×.

### 2.4. Microbubble Lonidamine Loading Quantification

The quantification of LND loading was performed as previously described [6]. In brief, all quantifications used a Dionex Ultimate 3000 HPLC system (ThermoFisher, Waltham, MA, USA) attached to a Thermo Orbitrap mass spectrometer. An XBridge C18 column (4.6 mm × 150 mm, 3.5 μm; Waters) was used for all separations. Freeze-dried sample vials were reconstituted in methanol, vortexed for 15–20 s, filtered with a syringe filter, and analyzed by HPLC analysis. The mobile phase was composed of 50% solvent A (0.1% formic acid in water) and 50% solvent B (0.1% formic acid in acetonitrile) by the isocratic method at a flow rate of 0.2 mL/min [29]. Each injection ran for 17 min at a volume of 5 μL with the compartment temperature at 4 °C and the column temperature at 30 °C.

### 2.5. Cell Line and Reagents

A well-characterized HNSCC cell line, CAL27 (ATCC, Manassas, VA, USA), was maintained in growth media consisting of Dulbecco’s modified Eagle’s medium (DMEM), High Glucose, Gluta MAX™ (Gibco™, Waltham, MA, USA) supplemented with 10% fetal bovine serum (FBS; Corning, NY, USA) and 1% penicillin–streptomycin (ATCC, Manassas, VA, USA) and kept at 37 °C in 5% CO_2_.

### 2.6. Implantation and Tumor Growth

Animal experiments were carried out in accordance with Thomas Jefferson University’s Institutional Animal Care and Use Committee (IACUC; approved 15 June 2020). HNSCC tumors were generated in athymic nude mice (The Jackson Lab, Bar Harbor, ME, USA) (split evenly by gender) subcutaneously on the right flank with 5 × 10^5^ CAL27 cells (ATCC, Manassas, VA, USA) and 100 μL matrigel (Corning, NY, USA). Once tumors reached 100 mm^3^, animals were randomly assigned to control and treatment groups with metformin pre-treatment (OM) (Table 1). Animals were monitored twice per week using digital caliper measurements until sacrifice was required (tumor mass > 10% body weight or animal showing signs of distress).

### 2.7. In Vivo Acoustic Analysis

Each microbubble group listed in Table 1 received a 0.1 mL bolus injection of agent followed by a 0.05 mL saline flush with ultrasound triggering at the tumor. Continuous B-mode and contrast mode imaging was acquired using a 10L4 transducer and an Acuson Sequoia (Siemens Healthineers, Issaquah, WA, USA) ultrasound scanner. Following peak enhancement, a 4-second destructive pulse was employed to trigger and destroy microbubbles within the tumor environment (MI = 1.4). This was followed by 10 s of low MI imaging to visualize microbubble reperfusion (MI = 0.12) and repeated for 75 s.

### 2.8. Treatment Administration

The 61 tumor-bearing mice were randomly assigned to the groups listed in Table 1. Therapy response was compared with and without O_2_ microbubble pre-sensitization. Animals in groups receiving metformin pre-treatment had it added to their drinking water at a concentration of 1 mg/mL [21,30,31]. The mice received 300 mg/kg/day on average. Following the same protocol previously reported for injectable anesthetics [6,24], animals from each group were anesthetized using a mixture of ketamine (75 mg/kg) and acepromazine (1 mg/kg). SE61 groups received microbubbles through a 24-gauge tail vein angiocatheter. Following microbubble administration, animals receiving radiation therapy were placed 40 cm from the radiation source and covered with 4 mm lead shielding, exposing the right flank and tail. Immediately following ultrasound triggering, animals received 5 Gy (for an estimated 25% tumor inhibition) using an X-RAD 320 biological irradiator (Precision X-Ray, Madison, CT, USA) with a beam quality of 320 kV with two different added filtrations. Overall, 44 animals received 5 Gy with a 2 mm aluminum filter (F1), and 17 animals received 5 Gy with a 1.5 mm aluminum, 0.25 mm copper, and 0.75 mm tin filter (F2). These filters corresponded to radiation dose rates of 3.59 and 1.36 Gy/min, respectively. Following treatment, animals were monitored until sacrifice was required per the criteria mentioned above.

### 2.9. Data Analysis

Significant differences between acoustic properties were determined using a one-way ANOVA. Tumor volumes for each animal were plotted against time after treatment. A simple survival analysis was conducted in Prism (GraphPad Software, San Diego, CA, USA) using Kaplan–Meier curves from the day of treatment until the day each animal was sacrificed. To analyze differences between groups and filters, log-rank tests were used. Differences in growth rate parameters were established using a Generalized Estimating Equations (GEE) model of exponential growth for robust standard error estimation accounting for the autoregressive correlations among the longitudinal measures within animals. A significance level of α = 0.05 was used, and statistical analysis was performed in Prism and SAS v 9.4 (SAS Institute, Cary, NC, USA), with plots of the models created in R (Wirtschaftsuniversität, Vienna, AT, USA).

## 3. Results

### 3.1. Microbubble Lonidamine Loading and Physical Characterization

**Figure 1:**
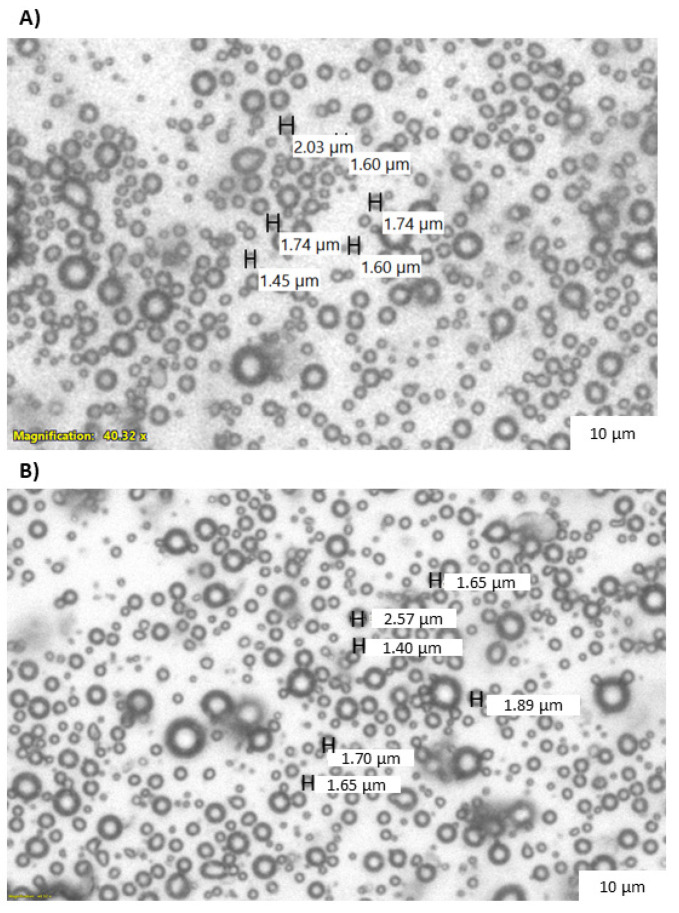
Light microscopy images of (**A**) SE61O_2_ and (**B**) SE61O_2_/LND.

Following characterization with HPLC, LND encapsulation within the microbubble showed an average of 25.7 ± 1.5 μg/mL, with a total of 705.7 ± 80.8 μg LND encapsulated per batch. Though the encapsulation efficiency was low (1.71%), it was able to surpass the minimum required loading of 4.8 μg/mL based on the minimum effective LND dose in tissue [28,32]. The population and size distribution LND microbubbles have been previously reported, at an average of (2.7 ± 0.2) × 10^9^ MB/mL [6]. Light microscopy images showing the visual representation of the microbubble sizes to display visible changes in the microbubble morphology after adding LND are shown in Figure 1. 

### 3.2. Microbubble Imaging In Vitro and In Vivo

**Figure 2:**
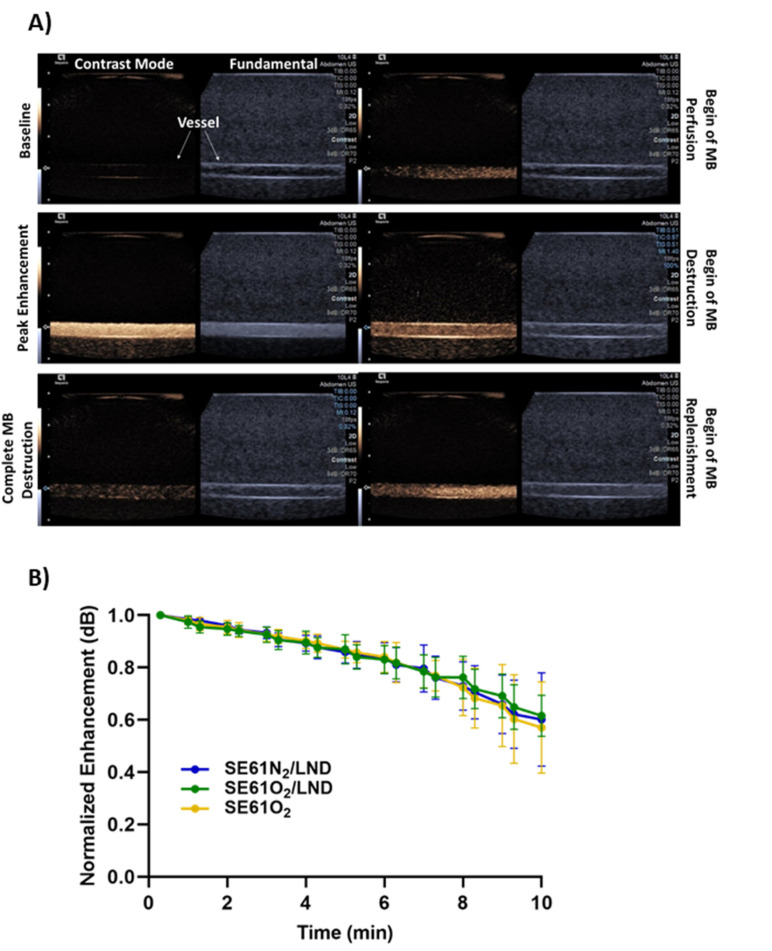
(**A**) Illustrative ultrasound images of SE61O_2_/LND in a closed-loop flow phantom showing contrast mode (**left**) and fundamental (B-mode) (**right**). Image depth markers correspond to 0.3 cm increments with the flow direction from right to left. (**B**) Microbubble stability curves of SE61O_2_/LND (green), SE61O_2_ (yellow), and SE61N_2_/LND (blue) at a non-destructive MI (MI = 0.12). No statistically significant differences among formulations were observed in vitro (*p* = 0.99).

The ability to non-invasively destroy SE61 microbubbles with or without LND and with or without O_2_ was confirmed in a pulsatile flow phantom setup, where agents were insonated in a single plane with higher intensity ultrasound pulses (MI = 1.4) (Figure 2A). All samples showed strong signals within the lumen and remained stable for around 10 min. Stability curves were constructed with the normalized enhancement of each region of interest in the contrast imaging plane every 30 s (shown in Figure 2B). All samples had 80% retained signal for over 7 min. No significant differences were observed in agent stability with or without LND or between O_2_ and N_2_ cores (*p* = 0.99).

**Figure 3:**
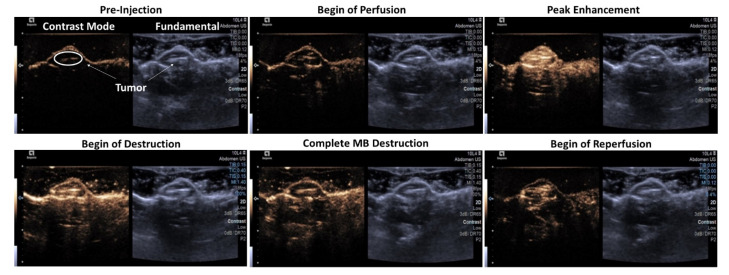
Illustrative ultrasound images of SE61O_2_/LND in an HNSCC tumor model generated in immunocompromised mice in dual B-mode/cadence pulse sequencing mode. Images show pre-injection of microbubbles (MB), start of MB perfusion, peak enhancement post-injection, start of MB destruction, complete MB destruction, and start of reperfusion (image depth corresponds to 0.3 cm increments). The left side of the display is the nonlinear imaging mode (contrast), and the right is the conventional B-mode (grayscale imaging).

Imaging and tolerability in the HNSCC tumor model in vivo showed that all microbubbles samples, whether oxygenated, charged with nitrogen, and/or drug-loaded (SE61O_2_, SE61O_2_/LND, and SE61N_2_/LND), were well tolerated following intravenous injection. These agents demonstrated perfusion into the tumor environment with strong enhancement on contrast-specific imaging (Figure 3). Following administration, at peak enhancement, a destructive pulse sequence was employed to destroy microbubbles within the tumor (MI = 1.4) followed by 10 s of low MI imaging to visualize reperfusion (MI = 0.12). This flash-replenishment sequence was repeated for 75 s, confirming the ability to destroy these agents locally as well as their stability in the systemic circulation (Figure 3). Images show the time course of SE61O_2_/LND from prior to injection (i.e., baseline) over complete perfusion to complete destruction and reperfusion after the destructive pulses.

### 3.3. Therapy Experiments

**Figure 4:**
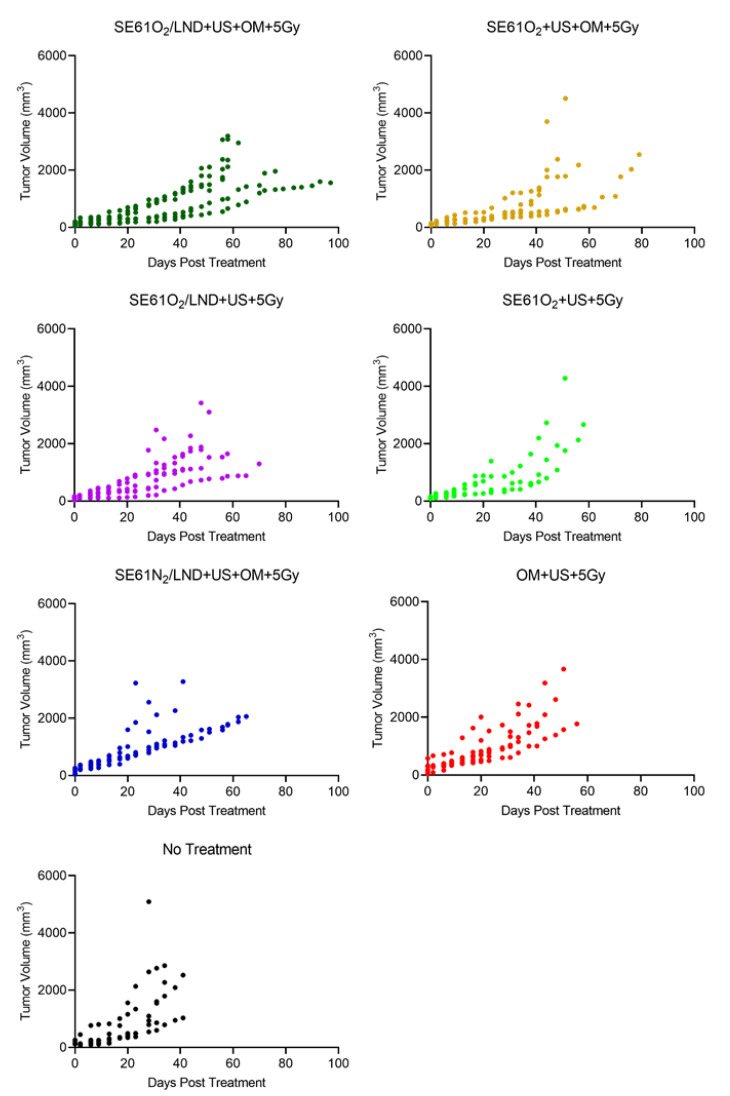
Tumor growth over time per animal for each group showing raw tumor volumes (mm^3^) from the day of treatment until sacrifice was required. Results show the influence of ultrasound (US), SE61 microbubbles, radiation therapy (5 Gy), gas (oxygen or nitrogen), and tumor mitochondrial respiration inhibitors (metformin (OM) and lonidamine (LND)) on an HNSCC tumor model.

**Figure 5:**
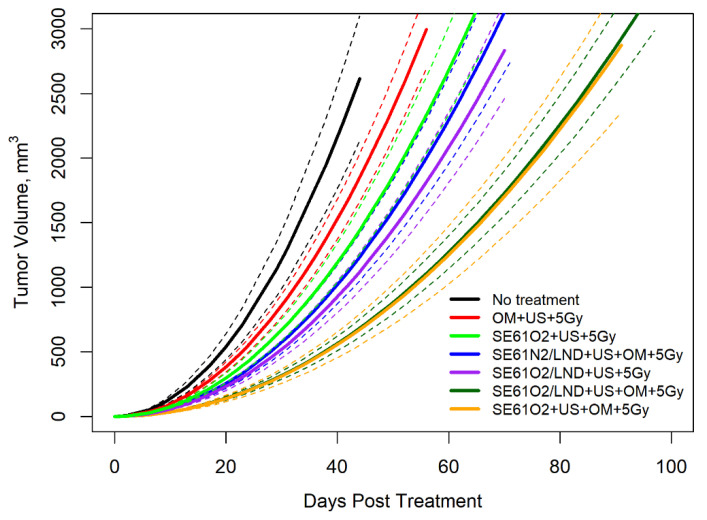
Tumor volumes plotted against time with fitted exponential growth curves showing tumoral response to therapy, with 95% confidence bands shown as dashed lines. Tumor volumes are plotted to the day of treatment to show the influence of ultrasound (US), SE61 microbubbles, radiation therapy (5 Gy), gas (oxygen or nitrogen), and tumor mitochondrial respiration inhibitors (metformin (OM) and lonidamine (LND)).

Therapeutic enhancement evaluated in an HNSCC tumor model is shown in Figure 4 comparing tumor growth over time. The results demonstrate that despite some expected variability due to differences in tumor size and growth rate, the experimental group that received SE61O_2_/LND with OM pre-treatment followed by 5 Gy delivered with F1 had improved tumor growth control compared to animals in the SE61O_2_ + US + 5 Gy, OM + US + 5 Gy, and the no treatment groups. These results were further confirmed after fitting to a regression model curve, shown in Figure 5. Both O_2_ microbubbles (shown in dark green and orange) receiving OM pre-treatment and 5 Gy showed a statistically significant improvement in tumor control relative to the other controls (*p* < 0.01) other than N_2_ microbubbles (blue in the plot) and LND microbubbles without OM (purple in the plot). For LND-loaded bubbles treated with ultrasound and 5 Gy radiation, the presence of O_2_ (versus N_2_) showed approximately 20 days of improvement in tumor control, although this was not statistically significant (*p* = 0.11). However, no differences were observed in tumor growth volumes between animals receiving OM pre-treatment, 5 Gy radiation, and O_2_ microbubbles with or without LND (*p* = 0.83) and SE61O_2_/LND with or without OM (*p* = 0.17). These results indicate that while the addition of each mitochondrial tumor respiration inhibitor alone improves tumor control, no additional benefit is provided by combining multiple inhibitors.

**Figure 6:**
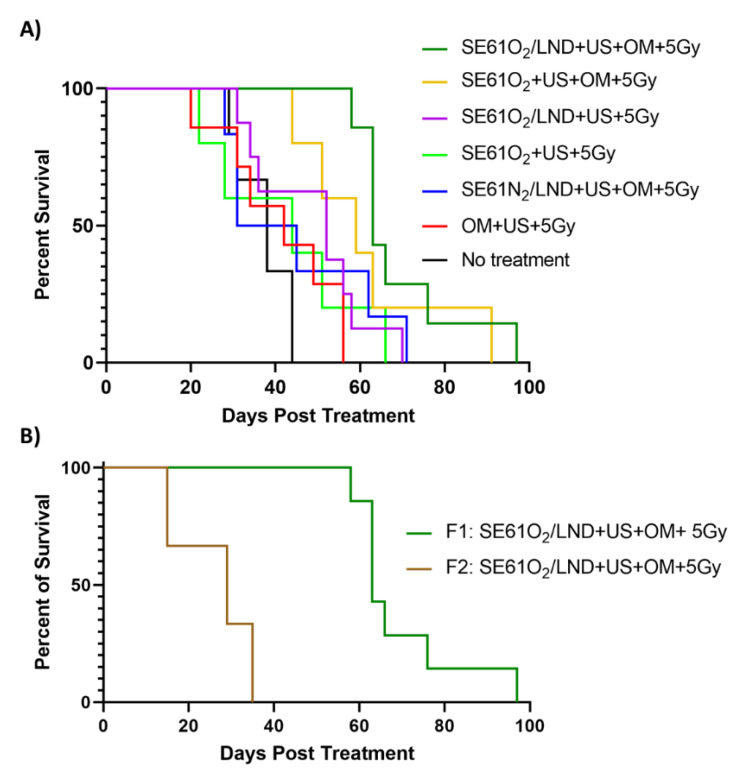
Survival proportion of animals following treatment until day of sacrifice. (**A**) Animal survival of all groups; treated groups received radiation with Filter 1 (F1; 3.59 cGy/min). (**B**) A comparison of the experimental group (SE61O_2_/LND) with ultrasound triggering (+ US) and metformin pre-treatment (OM) that received radiation with Filter 1 (dark green plot) and animals treated with the same treatment but that received radiation with Filter 2 (F2; 1.36 cGy/min) (brown plot).

The influence of radiation dose rate on animal survival is shown in Figure 6A,B. Similarly, animals treated with O_2_ LND-loaded microbubbles, OM pre-treatment, and 5 Gy radiation (dark green in the plot) showed an improvement in median animal survival (63 days) compared to all control groups (*p* = 0.0064), except for animals that received O_2_ microbubbles with OM pre-treatment and 5 Gy of radiation (orange in the plot) (*p* = 2873) (Figure 6A). Additionally, the experimental group (dark green plot) receiving SE61O_2_/LND with OM pre-treatment had a probability of survival of 86% at 58 days post-treatment, which was statistically significant compared to all other groups (*p* = 0.003). Interestingly, animals treated with SE61O_2_/LND with OM pre-treatment showed an improvement in animal survival compared to animals treated with SE61O_2_/LND without OM pre-treatment (*p* = 0.0108). However, there was no difference in median animal survival for animals that received O_2_ microbubbles, metformin pre-treatment, and 5 Gy radiation (without LND), reaching 59 days (*p* = 0.29). Additionally, animals that received SE61O_2_/LND and OM pre-treatment followed by 5 Gy delivered with F1 (3.59 cGy/min) showed an improved animal survival (*p* = 0.0008), with a median survival of 63 days, when compared to animals that received the same treatment with 5 Gy delivered with F2 (1.36 cGy/min), reaching 29 days (Figure 6B). This demonstrates that while the platform provides a significant improvement in tumor control with co-encapsulated LND and OM, a relatively high radiation dose rate is still required given the limited duration of oxygenation.

## 4. Discussion

Disrupting ultrasound-sensitive microbubbles containing oxygen locally may overcome tumor hypoxia prior to radiotherapy. Earlier in vivo studies by our group demonstrated the feasibility of disrupting oxygen microbubbles within a murine breast tumor model and a model of metastatic breast cancer in the brain, which raised the mean tumor pO_2_ to as much as 20 mmHg. This increase in oxygenation significantly improved tumor control and animal survival [24,26]. The sensitization capability of oxygen-loaded microbubbles before radiation therapy has also been proven by others [33,34,35,36]. Other groups explored the ability to enhance the efficacy of treatment by decreasing tumors’ oxygen consumption with mitochondrial oxidative phosphorylation inhibitors or agents to depress the expression of programmed death-ligand 1 (PD-L1) in tumors [37,38,39,40,41].

More recently, our group investigated the ability to deliver oxygen in combination with a pharmacological tumor mitochondrial respiration inhibitor, LND, to HNSCC tumors to prevent cells from upregulating oxidative phosphorylation, which in turn extended the duration of oxygenation in tumors [6]. That work demonstrated that O_2_, when delivered along with LND, raised the pO_2_ by nearly 30 mmHg in HNSCC solid tumors, with levels remaining elevated for up to 5 min [6]. This work showed the therapeutic efficacy of co-delivering O_2_ microbubbles with mitochondrial respiration inhibitors, namely LND or metformin. The stability of this platform was confirmed in vitro in a pulsatile flow phantom. Stability curves showed that all samples, independent of gas and/or drug content, had 80% retained signal for over 7 min (cf., Figure 2).

Imaging and tolerability examined in vivo revealed that all microbubble samples, whether oxygenated, charged with nitrogen, and/or drug-loaded (SE61O_2_, SE61O_2_/LND, and SE61N_2_/LND), were well tolerated following intravenous injection, with no adverse reaction in any animal. The ability to improve tumor control and survival by sensitizing HNSCC tumors to radiotherapy was explored in this model. Regression modeling demonstrated that both O_2_ microbubbles (with and without LND) receiving OM pre-treatment and 5 Gy showed a statistically significant improvement in tumor control relative to the other controls (*p* < 0.01), except for the N_2_ microbubbles (*p* = 0.11) and LND microbubbles without OM pre-treatment (*p* = 0.17). This demonstrates that both metformin alone (light green versus orange growth curves in Figure 5) and LND alone (light green versus purple growth curves in Figure 5) had an added effect on radiosensitization in this model. We attribute the more pronounced effects of metformin to the higher intratumoral concentrations due to its superior bioavailability (>50% orally [21]). Notably, the addition of multiple mitochondrial respiration inhibitors (orange versus dark green growth curves in Figure 5) did not provide additional benefits, presumably due to both targeting similar mechanisms for inhibiting aerobic glycolysis.

Microbubble destruction (without O_2_) has been shown to increase radiosensitivity by inducing cell apoptosis due to increased ceramide production induced by the shear stress generated from inertially cavitating microbubbles [24,42,43,44]. These findings are reflected by the improved radiosensitization of animals receiving N_2_ microbubbles (although tumor control was further improved with the addition of O_2_). Additionally, we hypothesize that the limited improvement in tumor control with the addition of LND is due to the relatively low drug concentration (~2.55 µg/injection, considerably less than prior preclinical experiments using intraperitoneal injection [27]) and due to the application of radiation over a short enough duration whereby both O_2_ agents provided sufficient tumor oxygenation based on prior work [6]. An interesting finding in this work was the influence of radiation dose rate on therapeutic benefits, shown by delivering 5 Gy with two different filtrations (3.59 cGy/min vs. 1.36 cGy/min). Animals placed in our experimental group receiving SE61O_2_/LND, OM pre-treatment, and 5 Gy delivered with F1 showed improved tumor growth control and survival with a median survival of 63 days when compared to animals that received the same treatment with 5 Gy with F2 (29 days; *p* = 0.0008) (Figure 6B). This demonstrates that while the platform provides an improvement in tumor control, a relatively high radiation dose rate is still required given the limited duration of oxygenation currently available with this microbubble platform.

Though this study produced encouraging results, several limitations exist. The therapeutic efficacy of this microbubble platform was only evaluated in immunocompromised mice, which prevents an analysis of the immune system’s role in treatment response. The experimental goal of this study was to show tumor burden and animal survival; for this reason, we have not yet analyzed acute cellular response to radiation therapy with SE61 microbubbles. Future work will focus on immunocompetent animals to better assess the clinical relevance of this platform. We also anticipate expanding this work to other solid tumors easily accessible to ultrasound and currently treated with radiotherapy such as breast cancer, prostate cancer, and soft tissue sarcoma. Additionally, the radiation dose rate should be better refined to reflect changes in tumor oxygenation duration with the addition of LND. Finally, to overcome the relatively low drug concentration of LND, future work will focus on optimizing the LND dose delivered.

## 5. Conclusions

This work demonstrated that the co-delivery of O_2_ via ultrasound-sensitive microbubbles combined with a tumor mitochondrial respiration inhibitor improved radiosensitivity and animal survival in an HNSCC model. Additionally, this work highlights the importance of dose rate in the context of the limited duration of oxygenation currently available with this microbubble platform.

## Figures and Tables

**Table 1:** Treatment groups.

GROUP	TREATMENT	ULTRASOUND	5 GY	OM	n:(F1)	n:(F2)
**1**	SE61O_2_/LND	Yes	Yes	Yes	7	3
**2**	SE61N_2_/LND	Yes	Yes	Yes	6	2
**3**	SE61O_2_	Yes	Yes	Yes	5	3
**4**	SE61O_2_/LND	Yes	Yes	No	8	3
**5**	SE61O_2_	Yes	Yes	No	5	3
**6**	OM	Yes	Yes	Yes	7	1
**7**	None	No	No	No	6	2

## Data Availability

Data will be made available on request.
